# Counteracting Radio-Resistance Using the Optimization of Radiotherapy

**DOI:** 10.3390/ijms21051767

**Published:** 2020-03-05

**Authors:** François Chevalier

**Affiliations:** 1 UMR6252 CIMAP, CEA-CNRS-ENSICAEN-Université de Caen Normandie, 14000 Caen, France; chevalier@ganil.fr; 2 LARIA, iRCM, François Jacob Institute, DRF-CEA, 14000 Caen, France; 3 ARCHADE Research Community, 14000 Caen, France

Radiotherapy is an essential component of cancer therapy and remains one of the most (cost-) effective treatment options available. Carbon ion radiotherapy (CIRT) promises to improve outcomes compared with standard of care in many cancers. The benefits of the better ballistics and higher efficiency of CIRT have been demonstrated in the last decade. However, although the better ballistic of protontherapy is already successfully used worldwide, hadrontherapy with carbon ions is underused and raises some concerns about likely side effects for patients. In addition, clinicians often observe in-field recurrence after CIRT. This indicates the presence of a subset of cancers that harbor an intrinsic resistance to CIRT. Several cellular mechanisms attenuating the efficacy of irradiation have long been known to occur, such as hypoxia, DNA damage repair, repopulation or cell cycle redistribution. Radiobiology research into tumor radioresistance has thus focused on targeting these mechanisms to increase radiotherapy effectiveness by, e.g., hypoxia modification, combination therapy with DNA damage repair and growth hormone inhibitors, or radio-enhancement components, such as metallic nanoparticles.

In this special issue of the *International Journal of Molecular Sciences*, “Counteracting Radioresistance Using the Optimization of Radiotherapy”, several studies have been published addressing some of the aspects related to tumor radio-resistance, with one research article [1], two communication articles [2,3], one review article [4] and one technical note [5].

In their study, Wang and coworkers [1] analyzed the radiation-enhancement effect of TiO_2_ nanoparticles co-exposed during irradiation with human lung cells. Nanoparticles or nanomaterials are now integrated into a large number of consumer products, including cosmetics, food, aerosols, tires, paints and electronic components. As a result, the population is largely exposed through all the conventional routes of contamination: ingestion, inhalation and skin. The dangerousness of these objects and their risks for human health are extensively studied in large toxicology programs, but, to date, the consequences of their presence during the time-course of radiotherapy has never been studied, especially the risk for healthy tissues. Given the frequency and quantity of TiO_2_ nanoparticles in numerous products, their dangerousness for healthy tissues during radiotherapy is of real interest. In this paper, the authors showed a clear radio-sensitizing effect of co-exposure of immortalized human lung cells with TiO_2_ nanoparticles and ionizing radiation.

In their case study, Darwis and coworkers [2] observed an enrichment of somatic mutations in *FGFR3* and *FGFR4* genes in the CIRT-recurrent tumor compared with the treatment-naïve tumor. The authors showed that the FGFR inhibitor could sensitize multiple cancer cell lines to carbon ions at 3 Gy (RBE: relative biological effectiveness), the daily dose prescribed to the patient. The sensitizer enhancement ratios were 1.66 ± 0.17, 1.27 ± 0.09, and 1.20 ± 0.18 in A549, H1299, and H1703 cells, respectively.

In their molecular and cellular study, Anakura and coworkers [3] compared the radiosensitivity to X-rays of several lung cancer cell lines according to the *EGFR* mutational status. The authors concluded that EGFR mutant cell lines might be more sensitive to low-dose- and low-fraction-sized-irradiation compared with WT cell lines.

In their review article, Thariat and coworkers [4] gave an up-to-date overview of the radiobiological and clinical aspects of hadrontherapy. This review articles deals with several aspects of hadronbiology and hadrontherapy in relation with treatment radio-resistance, such as proton therapy in tumor control; carbon ions and multi-ion therapy in hadron therapy; ultra-high-dose-rate flash proton therapy; hypoxia-induced radio-resistance; tumor microenvironment-induced immune tolerance; bystander effects in normal tissue tolerance in hadron therapy; biomarkers for the guidance of high-precision radiation therapy; and combined strategies to improve particle therapy.

The authors concluded with a positive prospective idea: individualized medicine would allow for an optimized delivery of radiation, such as ultra-high-dose-rate irradiation and multi-ion therapy, to provide tumor dose painting and normal tissue sparing due to better physical and biological properties. 

In their comprehensive detailed literature search, Matsui and coworkers [5] showed that two clonogenic assay parameters (SF2 and D10) have acceptable inter-assay precision (256 common cancer cell lines) and would facilitate the robust identification of biological profiles representative of cancer cell sensitivity to photons.

## References

[1] Wang Y., Sauvat A., Lacrouts C., Lebeau J., Grall R., Hullo M., Nesslany F., Chevillard S. (2020). TiO_2_ Nanomaterials Non-Controlled Contamination Could Be Hazardous for Normal Cells Located in the Field of Radiotherapy. Int. J. Mol. Sci..

[2] Darwis N.D.M., Nachankar A., Sasaki Y., Matsui T., Noda S., Murata K., Tamaki T., Ando K., Okonogi N., Shiba S. (2019). FGFR Signaling as a Candidate Therapeutic Target for Cancers Resistant to Carbon Ion Radiotherapy. Int. J. Mol. Sci..

[3] Anakura M., Nachankar A., Kobayashi D., Amornwichet N., Hirota Y., Shibata A., Oike T., Nakano T. (2019). Radiosensitivity Differences between EGFR Mutant and Wild-Type Lung Cancer Cells are Larger at Lower Doses. Int. J. Mol. Sci..

[4] Thariat J., Valable S., Laurent C., Haghdoost S., Pérès E.A., Bernaudin M., Sichel F., Lesueur P., Césaire M., Petit E. (2020). Hadrontherapy Interactions in Molecular and Cellular Biology. Int. J. Mol. Sci..

[5] Matsui T., Nuryadi E., Komatsu S., Hirota Y., Shibata A., Oike T., Nakano T. (2019). Robustness of Clonogenic Assays as a Biomarker for Cancer Cell Radiosensitivity. Int. J. Mol. Sci..

